# Measurement of local resolution in electron tomography

**DOI:** 10.1016/j.yjsbx.2019.100016

**Published:** 2019-11-25

**Authors:** J.L. Vilas, J. Oton, C. Messaoudi, R. Melero, P. Conesa, E. Ramirez-Aportela, J. Mota, M. Martinez, A. Jimenez, R. Marabini, J.M. Carazo, J. Vargas, C.O.S. Sorzano

**Affiliations:** a Biocomputing Unit, Centro Nacional de Biotecnologia (CNB-CSIC), Darwin, 3, Campus Universidad Autonoma, 28049 Cantoblanco, Madrid, Spain; b MRC Laboratory of Molecular Biology, Francis Crick Avenue, Cambridge CB2 0QH, United Kingdom; c U1196, Institut Curie, INSERM, PSL Reseach University, F-91405 Orsay, France; d Dept. Anatomy and Cell Biology, McGill Univ., Montreal, Canada; e Univ. San Pablo – CEU, Campus Urb. Monteprincipe, 28668 Boadilla del Monte, Madrid, Spain

**Keywords:** Local resolution, Electron tomography, Cryoem, Image processing

## Abstract

Resolution (global and local) is one of the most reported metrics of quality measurement in Single Particle Analysis (SPA). However, in electron tomography, the situation is different and its computation is not straightforward. Typically, resolution estimation is global and, therefore, reduces the assessment of a whole tomogram to a single number. However, it is known that tomogram quality is spatially variant. Still, up to our knowledge, a method to estimate local quality metrics in tomography is lacking. This work introduces *MonoTomo*, a method developed to estimate locally in a tomogram the highest reliable frequency component, expressed as a form of local resolution. The fundamentals lie in a local analysis of the density map via monogenic signals, which, in analogy to *MonoRes*, allows for local estimations. Results with experimental data show that the local resolution range that MonoTomo casts agrees with reported resolution values for experimental data sets, with the advantage of providing a local estimation. A range of applications of *MonoTomo* are suggested for further exploration.

## 1 Introduction

Electron microscopy has become in the last decade a strategic tool in structural biology (Cheng, 2018). Its main branches, single particle analysis (SPA) and electron tomography, present significant differences with respect to strategies for data acquisition and processing, as well as to the type of specimens they usually apply to. On the one hand, the former has led to the so-called resolution revolution and now enables near-atomic structure determination of proteins in solution (Kühlbrandt, 2014). On the other hand, the latter is the leading technique for 3D visualization of the architecture and molecular organization of cells and tissues in their physiological context (Beck and Baumeister, 2016, Jensen and Oikonomou, 2017). Although there are prospects to reach near-atomic resolution by means of subtomogram averaging, this technique still presents some resolution-limiting factors (Bharat et al., 2015).

Resolution aims at estimating up to which degree features can be reliably identified in the reconstruction. Unfortunately, there is not an universal definition of resolution, the most spread one being related to the size of the smallest detail that the map presents above the noise level. For a review in depth of resolution estimations see (Sorzano et al., 2017). In this work we will focus on estimating the highest and reliable local frequencies that can be distinguished in electron tomograms, this measure will be named local informational content or simply local resolution. Stated in more precise terms: (1) detecting the frequency for which noise and signal cannot be distinguished any longer and, (2), performing this task locally, voxel per voxel. We realize that this frequency could have easily been referred to as “local resolution”, and, indeed, we -and others- do so in the context of a typical SPA workflow using *MonoRes* (Vilas et al., 2018) or *ResMap* (Kucukelbir et al., 2014). However, the degree of signal and noise levels in a tomogram is much wider than in SPA.

Moreover, while in SPA the workflow to calculate resolution is well established, in electron tomography it is less straightforward. The Fourier Shell Correlation (FSC) (Harauz and van Heel, 2001) is considered as the standard resolution measure in SPA. This resolution metric measures the cross-correlation at different frequencies between two maps being, in this sense, a self-consistency measurement. Resolution is determined when the FSC (cross-correlation curve) drops below a specific threshold (Rosenthal and Henderson, 2003). This method was adapted to electron tomography by splitting the tilt series in two halves (one with the even projections and the other with the odd projections). The result consists of two tomograms, called odd and even, respectively. Thus, the FSC can be calculated with these two halves (Cardone et al., 2005), and it is commonly referred to as FSCe/o. Alternatively, still another resolution method, called Noise-compensated Leave One Out (NLOO), can be used to measure the resolution of tomograms (Cardone et al., 2005). This method reconstructs the tomogram with all the tilt series images except one, and then re-projects the reconstructed tomogram in the direction of the excluded image, determining the FSC between the reprojection and the excluded image. When the angular sampling of the tomogram is small enough, FSCe/o and NLOO cast similar results (Cardone et al., 2005). Nevertheless, the most reliable resolution value is provided by the NLOO method. Another interesting approach, although of limited spread, uses the FSC with a conical constraint (Penczek and Frank, 2006). Following this directional strategy, a new measurement called conical-FSCe/o was recently introduced with the aim of analyzing resolution anisotropy (Diebolder et al., 2015). The idea is to compute the FSCe/o along a discrete number of directions covering the whole projection sphere, analyzing then the variability of resolution. For a deep review and information about the measurement of resolution in tomography see (Kudryashev, 2018, Fernandez, 2012).

These methods allow the estimation of the resolution of tomograms. However, they are global measurements that reduce the quality of the whole tomogram to a single number. Similarly to what has been shown in SPA, the quality of a reconstruction is also locally dependent (Cardone et al., 2005, Cardone et al., 2013), though for other reasons than in SPA where the link to structural flexibility is obvious. In tomography the existence of different local resolution values in a tomogram may be linked to: sample informational content, sample quality, local defocus, ice thickness, or artifacts coming from the missing wedge among other factors. In this work we have analyzed the issue of detecting local variations in the informational content of a tomogram, developing a novel approach to it. The new algorithm is called *MonoTomo*, and it allows us to determine the local resolution in the reconstructed tomograms. For this purpose, the local resolution method that we developed for SPA, *MonoRes* (Vilas et al., 2018) was modified to deal with large volumes (tomograms) and their properties, as the corresponding noise statistics, in relatively short computational times. With this aim, we propose two approaches to estimate the amount of local noise: the set of micrographs is split in odd and even images, or the set of frames of each movie is split in two sets. Both approaches allow us to reconstruct two tomograms. The difference between them produces a tomogram composed only by noise. This noise tomogram then allows us to compare at different frequencies if the local amplitude of each voxel is significantly higher than the local energy of the noise. As a consequence resolution is determined as the limit at which the amplitudes of noise and signal cannot be statistically distinguished.

## 2 Method

*MonoTomo* aims to determine the local resolution map for tomograms in the same way as 3D resolution maps do. It is accomplished by means of the *MonoRes* algorithm (Vilas et al., 2018), establishing hypothesis tests at different frequencies to determine if the local amplitude of the signal (structure) can be measured above the noise level (background). However, the measurement of local noise and signal requires adapting *MonoRes* for electron tomography. For the sake of self consistency, the latter algorithm is briefly explained highlighting the main differences with *MonoRes*.

### 2.1 Background on the MonoRes algorithm

*MonoTomo*, as well as *MonoRes*, uses the so-called monogenic signals (Felsberg and Sommer, 2001, Unser and Van De Ville, 2010) to achieve a local decomposition of the original electron density maps in terms of monogenic amplitude and phase, see Appendix. The density map is high pass filtered at several frequencies calculating its monogenic amplitude. Loosely speaking, the monogenic amplitude defines the local energy of each point in the image/map. In the case of SPA, and for the situation in which only the final map was provided, the use of a binary mask allows us to establish a frontier between structure (signal) and background (noise). In this way, for each voxel an hypothesis test is performed to determine if its local amplitude is significantly higher than the noise distribution (outside the mask). A local resolution value is assigned when this test fails consecutively twice to avoid false positives. The described procedure is carried out in a frequency sweep from low to high resolution. For a deep explanation see the Appendix or the original *MonoRes* publication (Vilas et al., 2018).

### 2.2 Noise estimation

*MonoRes*, and therefore *MonoTomo*, estimates locally the highest measurable frequency of a signal (structure) above the noise level. Hence, noise estimation is the critical step for resolution measurement. In SPA the characterization of noise is simpler because it is based on two facts: 1) there exists a clear border between noise and signal, so that a binary mask can discriminate between them, 2) the noise distribution can be considered spatially invariant.

Unfortunately, in electron tomography these two assumptions do not usually hold. The first condition is not fulfilled due to the fact that structure-noise distinction in a tomogram is virtually impossible since the whole tomogram may present structural details. Additionally, the second condition is normally broken because of the acquisition geometry, which induces spatial dependence (Turonova et al., 2016). Furthermore, the structure of the specimen itself modifies the local noise of the tomogram. To overcome these problems, *MonoTomo* requires as input two tomograms, which are reconstructed with two independent set of images of the same specimen, respectively. To do that, two approaches are considered.

The first approach uses the idea introduced by (Cardone et al., 2005) of splitting the set of tilt series images in two independent sets, named odd and even, containing images corresponding to alternative angles of the tilt series. Two tomograms (called odd and even) reconstructed with half of the images result from this strategy.

The second approach that we suggest to obtain two tomograms consists in taking the set of frames belonging to each movie, and split it in two subsets of frames (even and odd) obtaining, thus, two tilt series with the same angular sampling. The two tilt series are then built with the even/odd frames at each tilt angle. This second approach is better than the first one because both tomograms (odd an even) keep the same angular sampling of the tilt series, which warranties a better noise estimation. Note that this solution can only be used if movies are recorded at the microscope.

In both approaches, splitting micrographs or frames, a tomogram of noise is obtained by computing the difference between the reconstructed tomograms. This strategy of reconstructing two tomograms is similar to the one used in SPA of producing two half maps from which the noise is calculated as the difference between both halves (Kucukelbir et al., 2014, Vilas et al., 2018). The use of two tomograms solves the noise measurement problem and also avoids the need of using a mask. It must be noted that, in an strict sense, the odd and even tomograms obtained by either micrograph or frames splitting are not completely independent, because tomographic reconstructions make use of alignment parameters that are determined considering the whole set of images. Nevertheless, this dependence introduced is weak, specially considering that this substraction process is only done for a coarse estimation of the noise distribution.

### 2.3 Local resolution estimation

The two tomograms are used to determine a tomogram of noise by their difference. Moreover, a mean tomogram is calculated by averaging the two reconstructed tomograms, obtaining information from the whole tilt series. This tomogram is used in the filtering process at several increasing frequencies. The resolution range is provided by the user, and the frequency step is by default 5 Å. However, smaller steps can also be considered. In fact, it is an advanced parameter that the user can modify. To avoid the rippling effect in filtered maps each filter implements a smoothing by means of a raised cosine whose tail length is 0.01 rad/px in digital frequency. For each frequency the monogenic amplitudes of both tomograms (mean and noise) are calculated. An hypothesis test is performed to elucidate which voxels exhibit higher local monogenic amplitudes than noise, in an statistical sense (this test is explained below). If the local monogenic amplitudes of signal is higher than the local monogenic amplitude of noise, then that voxel has at least the resolution given by the filter cutoff frequency. In contrast, if the hypothesis tests fails two consecutive hypothesis test, the cutoff frequency of the first time that the test failed is assigned.

The noise tomogram obtained by odd and even difference normally presents a spatially non-uniform noise distribution which ought to be taken into account to perform the statistical analysis. In other words, the noise distribution depends on the position. To quantify the noise variation, the tomogram of noise (monogenic amplitude of noise) was divided in small cubes with 100×100×100 voxels. The monogenic amplitude of noise distribution was then calculated in each region. A noise threshold is obtained by computing the 95th percentile of the monogenic amplitudes of noise distribution in each cube. Thus, if a voxel of the monogenic amplitude of the mean tomogram has an energy higher than the respective threshold (hypothesis test), that voxel can be measured at the filtering resolution. To avoid possible discontinuities among cubes, the set of thresholds was regressed by B-splines. B-Splines are very robust under the existence of outliers providing smooth functions that fit the dataset (Jonic and Sorzano, 2011).

## 3 MonoTomo calibration

**Fig. 1:**
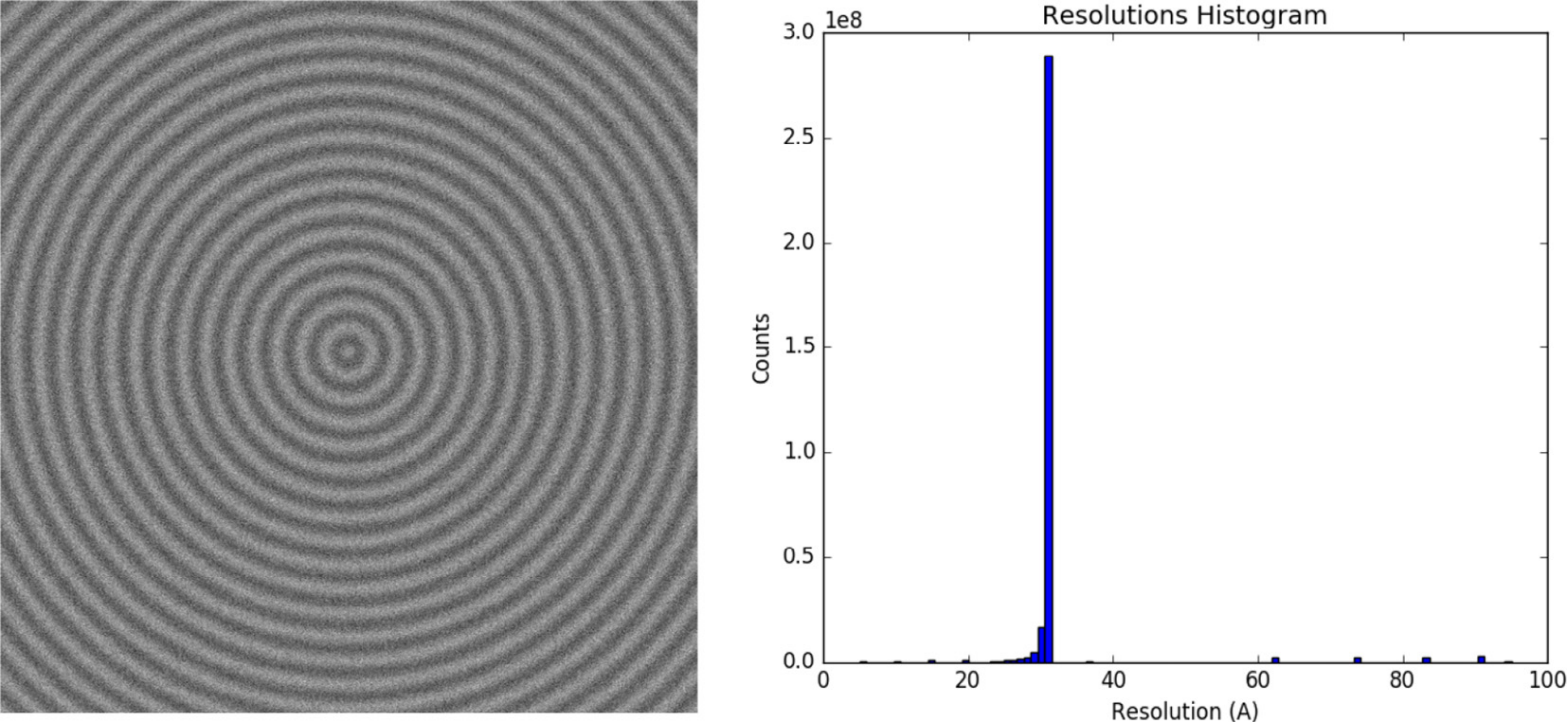
(left) Slice of the sinusoidal-spherical fringe pattern with wavelength of 30 Å. (right) Local resolution histogram for the corresponding tomogram.

To ensure that *MonoTomo* provides the proper estimation of local resolution, a test with synthetic data was used. The goal was to compute the local resolution of a tomogram for which the resolution was known. A fringe pattern was used with a pixel size of 1 Å/pixel, the pattern consists of a sinusoidal function with a wavelength of 30 Å, see Fig. 1. Note that the resolution of this map should ideally be well known and close to 30 Åin all positions. To achieve a realistic scenario, Gaussian noise with zero mean and standard deviation of 0.1 a.u. was added. Two tomograms were generated by performing two noise realizations added to two identical fringe patterns. Both tomograms will be the odd and even ones. Finally, *MonoTomo* was applied to both tomograms with the aim of estimating the local resolution. As was expected, *MonoTomo* provided a local resolution corresponding to the known wavelength of the fringe pattern immersed in noise. In Fig. 1 the resolution histogram is shown, with a clear peak at 30 Å. This test was also carried out with different wavelengths, recovering in all cases the corresponding wavelength.

## 4 Experimental results

*MonoTomo* was tested with experimental data sets from EMPIAR (10110 (Chang et al., 2017), 10115 (Swulius and Jensen, 2012), 10027 (Jiko et al., 2015) and 10164 (Schur et al., 2016)). For each EMPIAR entry, a tilt series was selected that was then aligned with IMOD (Kremer et al., 1996) based on fiducial markers. The tilt series were split in odd and even images (either on a micrograph or frames sense). That is, images corresponding to different tilt angles were alternatively distributed in two groups of images in the case of splitting micrographs, or the set of frames at each tilt angle was split in two groups in the approach of splitting frames; the frame splitting was only used with the first data set (EMPIAR 10164) because it was the only one with deposited frames in EMPIAR. Then, two tomograms were reconstructed using Tomo3D (Agulleiro and Fernandez, 2011, Agulleiro and Fernandez, 2015). These two tomograms were the input of *MonoTomo* for computing a tomogram with local resolution values. To analyze the range of resolution values that *MonoTomo* provides, the FSCo/e was also calculated. However, it is known that the FSC is sensitive to masking. As a consequence, the FSCe/o values are susceptible to give low resolution estimation when they are computed with the whole tomograms. Although the use of a mask to analyze the resolution in small regions of the tomogram is also common, and increases the FSCe/o values, in our test we have not used any mask. This is surely the reason why the FSCe/o values of the experimental cases are in the lower bound of the resolution range provided by MonoTomo. In all cases, the computational platform was a laptop with an Intel Core i7-8750H CPU at 2.20 GHz.

### 4.1 Experimental case 1

**Fig. 2:**
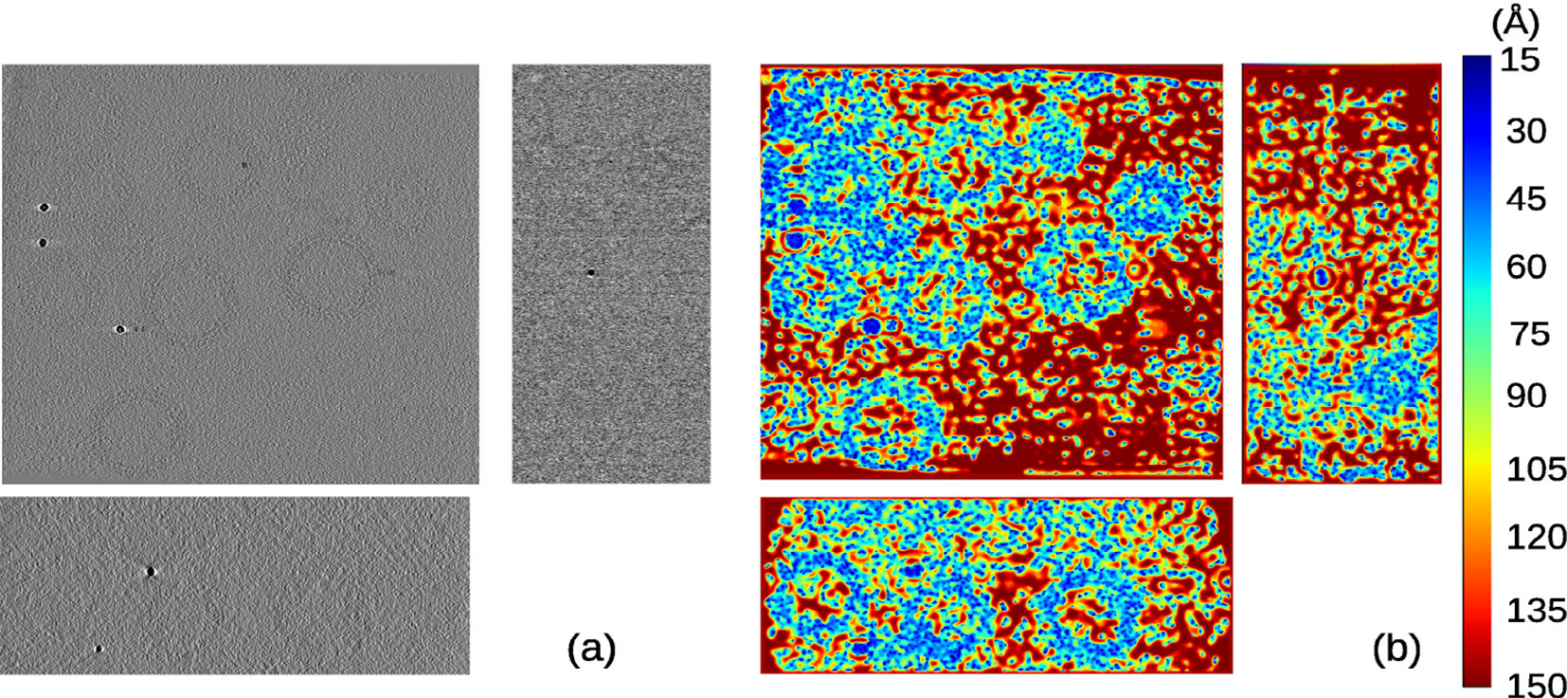
Slices along the x,y,z direction for the reconstructed tomogram (a) of the data set entry from EMPIAR 10164 and its corresponding local resolution slices (b).

The first experimental data set was taken from EMPIAR entry 10164 and contains a sample of HIV-1 virus (Schur et al., 2016). The tilt series was acquired with an angular sampling of 3 degrees from −60 to +60 degrees of tilt. The original movies had a pixel size of 0.675Å and they were aligned with MotionCor2 (Zheng et al., 2017). Note that this sample had a lower angular sampling, which implied a considerable loss of information by splitting the images tilt series in two sets (odd and even) because in that case, the odd/even set presented an angular sampling of 6 degrees. Thus, as an alternative for experimental acquisition with low angular sampling, the set of frames corresponding to each movie was split into even/odd sets to produce the even/odd tilt series at binning 2. The alignment of the original tilt series was done with IMOD based on fiducials and it was applied to the even/odd tilt series. Downsampling by a factor 4 was applied. *MonoTomo* used these tomograms to compute the local resolution. Results are shown in Fig. 2, providing values from 15 to 50 Åfor regions with noticeable structure. In Fig. 6 a histogram of local resolution values is shown. Note the FSCe/o value is 41 Å(at a threshold of 0.5).

### 4.2 Experimental case 2

**Fig. 3:**
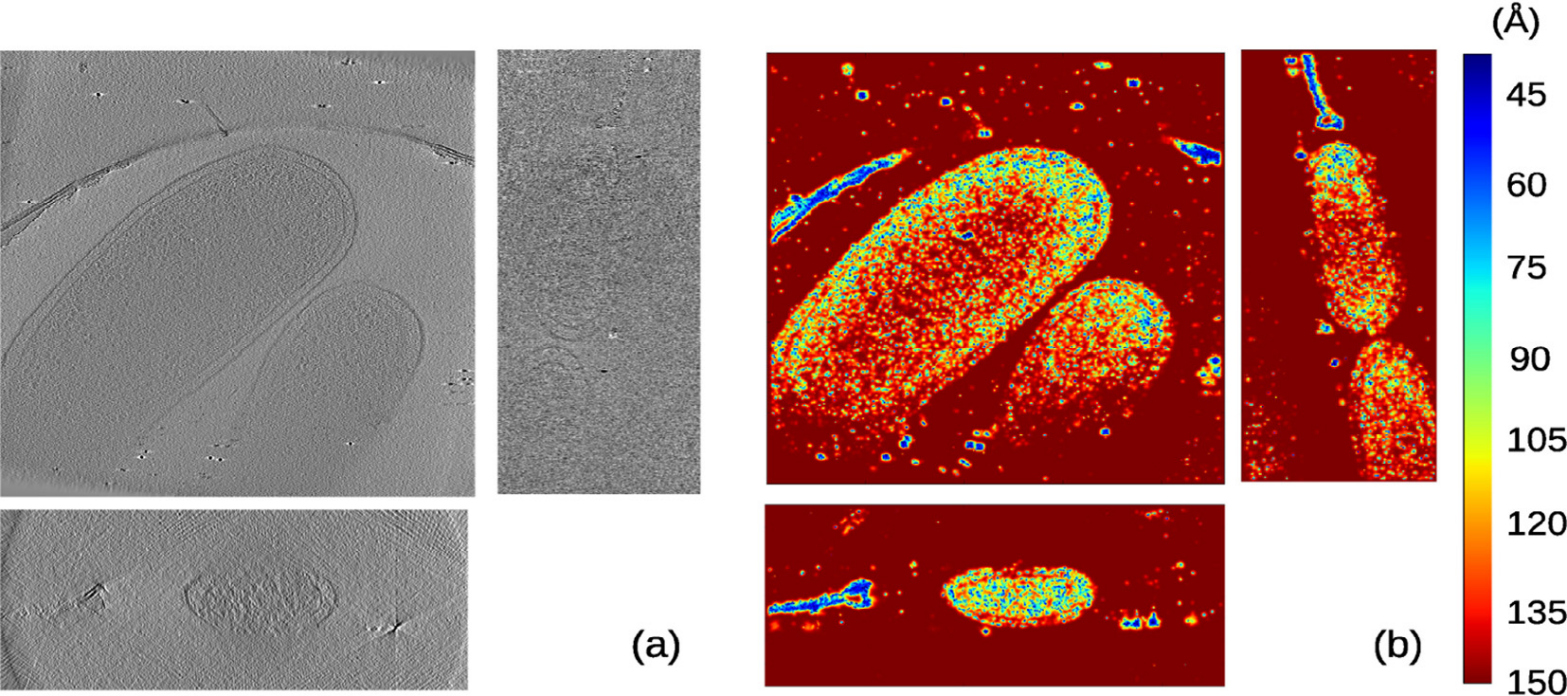
Slices along the x,y,z direction for the reconstructed tomogram (a) of the data set entry from EMPIAR 10110 and its corresponding local resolution slices (b).

*MonoTomo* was also applied to the data set from EMPIAR 10110 (Chang et al., 2017) in which *Vibrio cholerae* cells were reconstructed. This data set had an angular sampling of 1 degree with tilt angles from −60 to +60 degrees and a pixel size of 4.04 Å. To reduce the computational burden, the images were binned by a factor 4 getting a final pixel size of 16.16 Å/pixel. Then, the odd and even tomograms were reconstructed with dimensions of 928×960×400 voxel, and the local resolution was computed. *MonoTomo* results can be observed in Fig. 3, where several slices on the local resolution map are shown and compared with the corresponding slices of the density map. The resolution regions with clear structure is in the range between 40-120Å. Note how these latter values present a smooth transition between the very detailed regions and the background. In Fig. 6 a histogram of local resolution values is shown. Moreover, the FSCe/o was calculated and presents a value of 53.3 Å(at a threshold of 0.5). These results are compatible with those provided by *MonoTomo*.

### 4.3 Experimental case 3

**Fig. 4:**
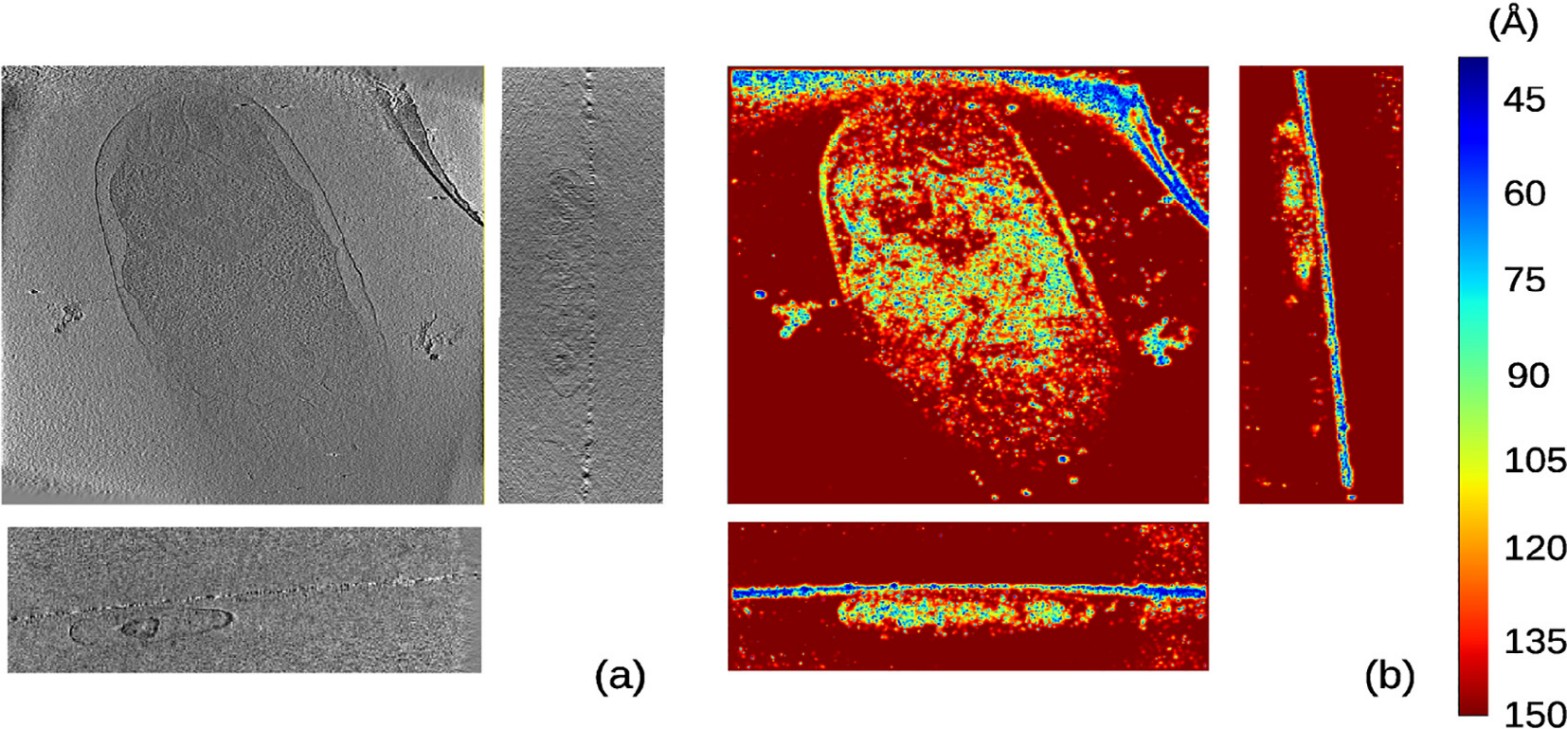
Slices along the x,y,z direction for the reconstructed tomogram (a) of the data set entry from EMPIAR 10115 and its corresponding local resolution slices (b).

The third experimental case was from EMPIAR entry 10115 (Swulius and Jensen, 2012) and contained *Escherichia coli* cells. Images of this dataset were acquired from −64 to  + 64 degrees of tilt angles with an angular interval of 1 degree and had a pixel size of 9.46 Å. As with the previous example, the micrographs were binned by a factor 2 to speed up the local resolution calculation, resulting in a pixel size of 18.92 Å and tomograms (odd/even) with size 1016×1096×200 voxel. In Fig. 4, *MonoTomo* results and the corresponding slices of this tomogram are shown. Thus, the tomogram presents an associated resolution in the range from 40 to 120 Å. In Fig. 6 a histogram of local resolution values is shown. In this case, the FSCe/o was also computed resulting in 155.2 Å(at a threshold of 0.5).

### 4.4 Experimental case 4

**Fig. 5:**
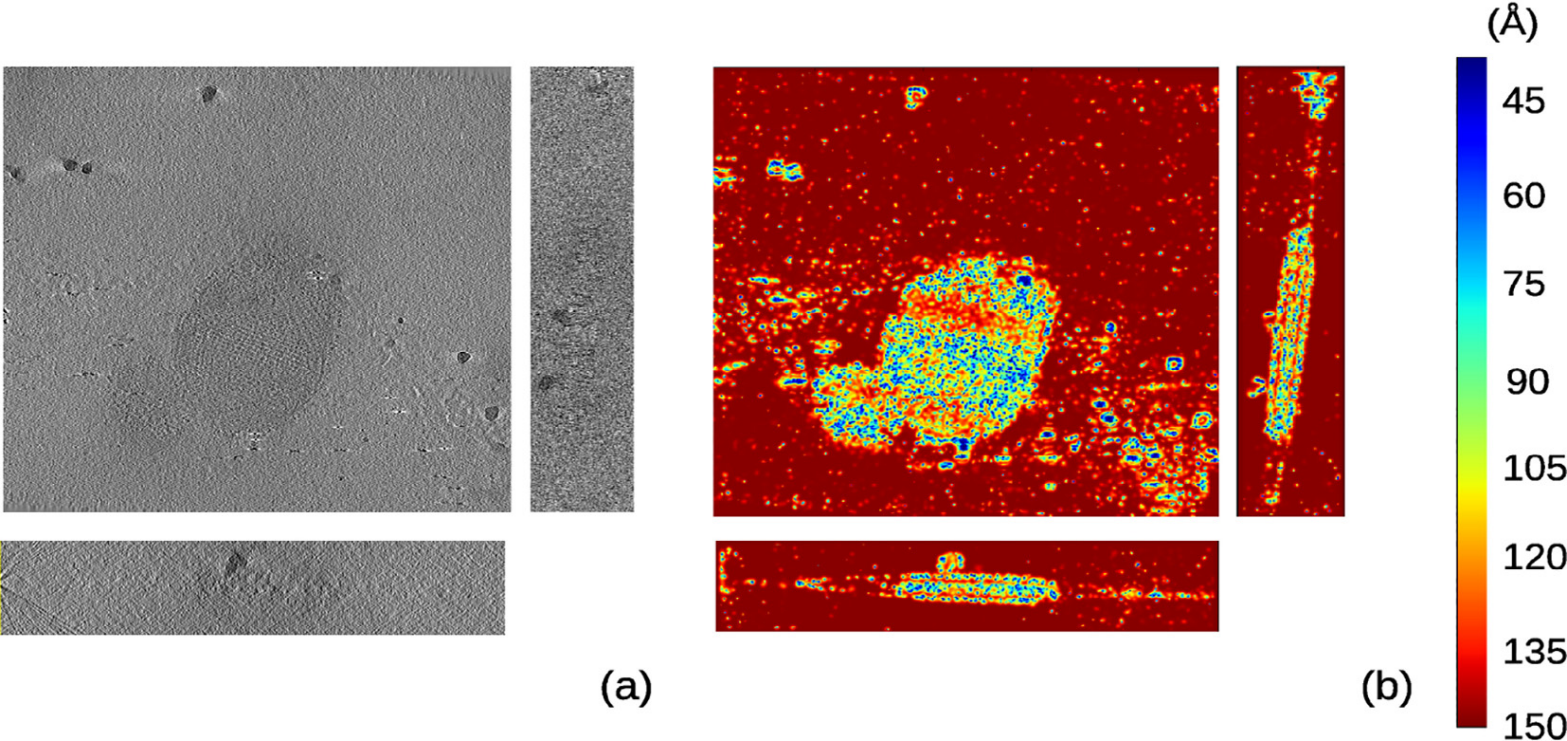
Slices along the x,y,z direction for the reconstructed tomogram (a) of the data set entry from EMPIAR 10027A and its corresponding local resolution slices (b).

**Fig. 6:**
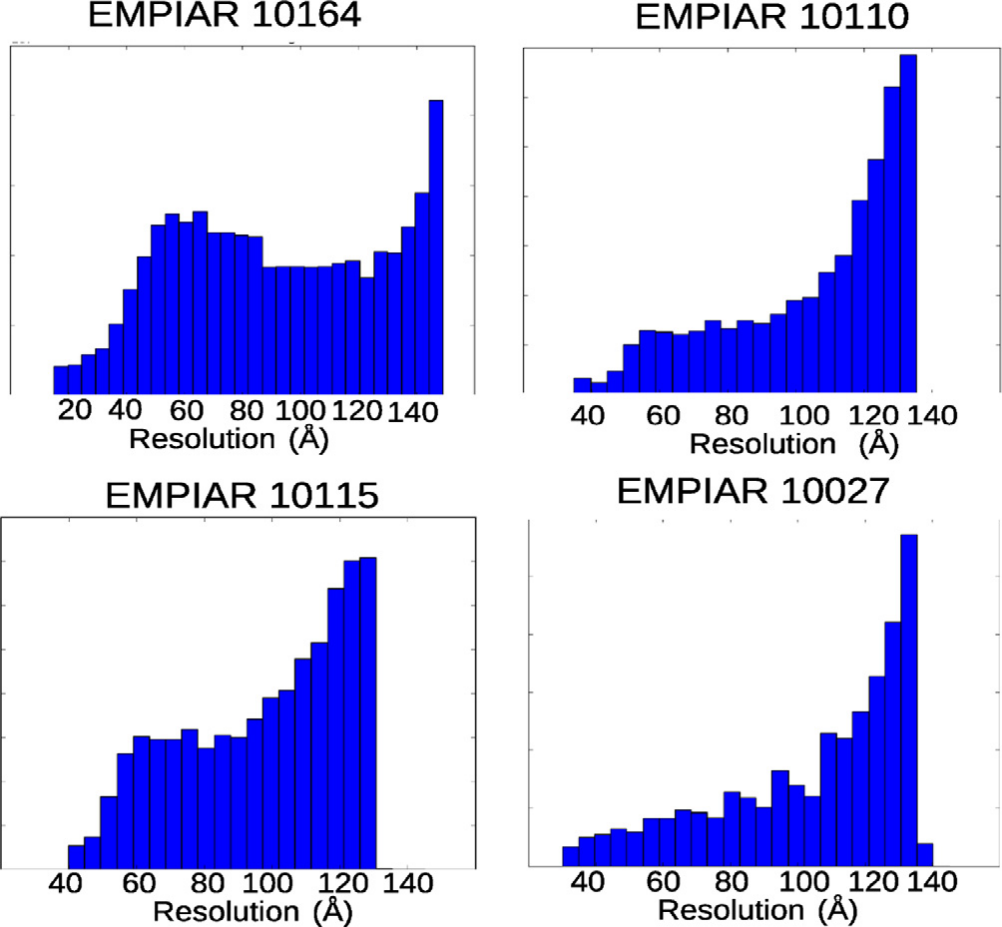
Histograms of the local resolution values provided by *MonoTomo* for reconstructed tomograms of the experimental dataset data sets from EMPIAR (10110 (Chang et al., 2017), 10115 (Swulius and Jensen, 2012), 10027 (Jiko et al., 2015) and 10164 (Schur et al., 2016)).

This last test made use of the experimental data set from EMPIAR 10027 (Jiko et al., 2015) in which the structure of intact bovine $F_{1}F_{o}$ ATP synthase in 2D membrane crystals was reconstructed. The tilt range was from −60 to +60 at an interval of 1.5 degrees, and the pixel size was 3.3Å. Micrographs were downsampled by a factor 4 getting a pixel size of 13.2 Å and split in odd and even sets to reconstruct the required tomograms with dimensions of 960×928×200 voxels. The results (Fig. 5) show that the resolution was in the range of 40–110 Åin regions with biological structure. In Fig. 6 a histogram of local resolution values is shown. For this experimental case, the FSCe/o was computed, getting a value of 53.2 Å (at a threshold of 0.5). The result of *MonoTomo* seems to be in agreement with the standard global resolutions.

## 5 Discussion

In this work we analyze the local informational content, i.e. the local resolution of a tomogram using an adaptation of a method we had previously proposed for estimating local resolution in SPA. The new method is called *MonoTomo* and is based on *MonoRes* (Vilas et al., 2018). Note that the informational content within a tomogram may be very spatially variant, with parts of the tomogram occupied by very well defined structural features (like organelles or even macromolecular complexes) and others that are very flat, with very few features. Naturally, the “signal” content in each of these parts is going to be very different, contrasting with the situation in Single Particle Analysis (SPA), in which basically we have protein against a relatively flat background (in other words, the signal is more or less constant and therefore the local resolution does not present high variation).

Among the very positive characteristics of *MonoTomo* we have that is almost fully automatic and only requires the specification of the resolution range of interest. In this way, just by using two tomograms, *MonoTomo* is able to determine a local resolution tomogram, by means of a frequency sweep and local hypothesis tests between the local amplitude of signal and noise in local regions. Results with experimental data show local resolution values compatible with the structures that tomograms present. In this regard, *MonoTomo* is able to determine without prior knowledge different regions of interest within the tomogram (ribosomes, cytoskeleton, membranes, virus, fiducials, or carbon borders among many others) which may be used for segmentation. Because of the absence of local resolution methods in the field of electron tomography, *MonoTomo* can only be compared to global values of resolutions. In particular, *MonoTomo* results for the tested experimental data set were compared to the FSCe/o values. However, it must be noted that the FSCe/o is mask dependent, and therefore, global FSCe/o produces lower resolution values than the FSCe/o obtained for specific areas of the tomogram (Cardone et al., 2005). The absence of mask in *MonoTomo* and its local character skip this pitfall, providing not only local values, but the full variation or gradient of local resolutions throughout the volume.

Regarding the limitations of *MonoTomo*, the main drawback is the very low SNR that tomograms present. *MonoTomo* makes use of the so-called monogenic signals (Vilas et al., 2018, Unser and Van De Ville, 2010, Felsberg and Sommer, 2001), which allow decomposing locally a function in terms of amplitude and phase. In our case, the function is the density map. Unfortunately, the reliability of the comparison between the local amplitudes of signal and noise is compromised when the angular step is large for two reasons. First, the SNR might be reduced as a consequence of the artifacts introduced by the angular gaps in the splitting micrographs approach. Second, the overlapping areas in Fourier space between the two tilt series (even/odd) obtained by splitting the images in the original tilt series is substantially reduced, which prevents a proper estimation of noise. These two problems complicate local estimations. In our tests, we have found that an angular sampling coarser than 2 degrees already introduces this sort of effects. To avoid that problem, we have proposed to split the frames corresponding to each image tilt in two halves (for those cases in which movies are recorded, of course). The problem with low angular sampling is thus avoided allowing us to calculate local resolutions as shown in the Experimental case 1. We also would like to highlight that *MonoTomo* is relatively fast in terms of computational times, it took around 15 min per tomogram in a normal laptop. In this regard, we recommend to bin the tomogram up to achieve sizes around 1000×1000×400.

Finally, we make the point that tomograms do not only have a locally changing signal component, since so many different structural features can be observed on them, but they also exhibit a large anisotropy, mostly due to the missing wedge. *MonoTomo*, following *MonoRes*, is not able to differentiate among the different directions converging onto a point on the tomogram. Fortunately, an extension of MonoRes (Vilas et al., 2018), called MonoDir is currently under evaluation (Vilas et al., under review), which will provide the bases for analyzing not only spatially local informational content, but also its variations along the different directions. We are, therefore, optimistic that in the middle term future local as well as directional estimation of local resolution will be able to be determined, which will provide still another unexplored new direction in tomographic quality assessment.

## 6 Conclusions

A fully automatic free-parameters method for determining local resolution of electron tomograms has been proposed. This new method provides an enriched information about resolution as a consequence of its local nature. It is achieved thanks to the use of *MonoTomo* algorithm based on the *MonoRes* core, which was carefully adapted to deal with spatially dependent noise. Moreover, *MonoTomo* was implemented in a very efficient manner to overcome the computational problems of dealing with large volumes of tomography. In addition, its fundamentals are conceptually simple, and up to our best knowledge, *MonoTomo* constitutes the first local resolution method in electron tomography. In this regard, it opens new possibilities with special incidence in the guidance of tilt-series alignment, selection of subvolumes for subtomogram averaging, or the use of local filters based on resolution measurements to enhance or sharpen the reconstructed tomogram. We also know that this method represents a first early step in the local resolution measurement in tomography and its applications. Finally, results with experimental data are in agreement with the expected values of resolution, and provide information about the local quality of the elucidated structures at a local level. *MonoTomo* has been carefully integrated in *Xmipp* (de la Rosa-Trevín et al., 2013) and *Scipion* v2.0 (de la Rosa-Trevín et al., 2016) and is publicly available under the *MonoTomo* protocol of Scipion.

## Declaration of Competing Interest

The authors declare that they have no known competing financial interests or personal relationships that could have appeared to influence the work reported in this paper.

## Figures and Tables

**Fig. 7:**
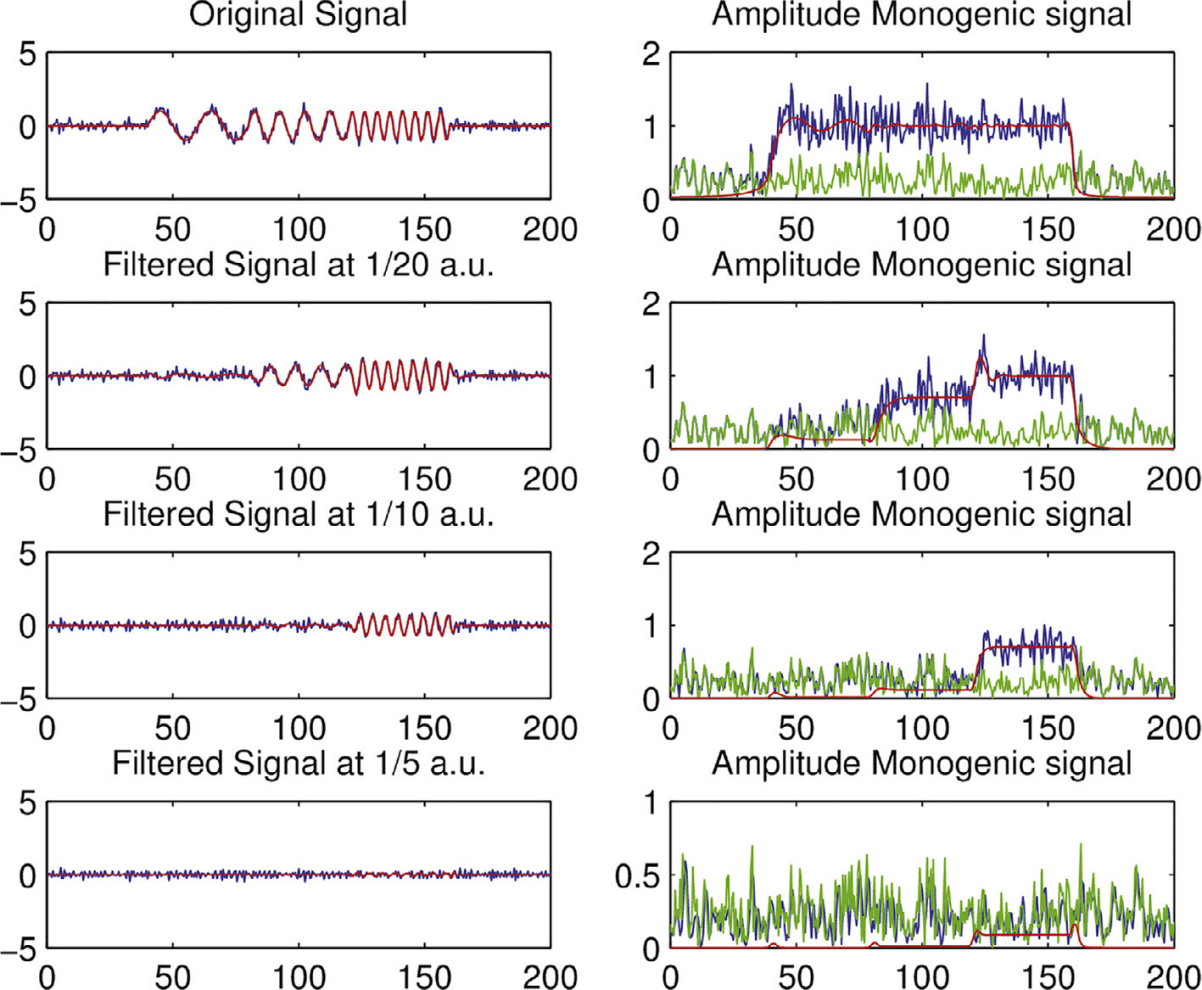
(left column-top) A signal composed frequencies of 1/20,1/10 and 1/5rad/px is high pass filtered at frequencies of 1/20,1/10 and 1/5rad/px (left-column). The corresponding monogenic amplitude is calculated and represented (right column). Note how the monogenic amplitude of signal and noise cannot be distinguished when the signal is high pass filtered at 1/20,1/10 and 1/5rad/px. As a consequence, the resolutions of 20, 10 and 15 a.u. are assigned to the corresponding interval.

## Appendix A Appendix

To understand *MonoTomo* algorithm it is necessary to understand the concept of monogenic signal. It provides an extension to multiple dimensions of the concept of analytic signal (Gabor, 1946). For the sake of simplicity, an unidimensional example will be considered for explaining such concept, which can then be extrapolated to multiple dimensions, see (Felsberg and Sommer, 2001, Unser and Van De Ville, 2010).

Let as consider a sinusoidal wave with frequency ω and amplitude A given by s(t)=Asin(ωt). It is known that the energy, *E*, of the wave is proportional to the square of the amplitude, it means $E={\mathrm{kA}}^{2}$, with a proportionality constant, *k*. The zeroes of s(t) are located at t=nπ/ω with *n* being an arbitrary integer. The instantaneous energy is defined as $s^{2}(t)$, however, the existence of zeroes does not mean that the wave lacks of local energy. For that reason, we are interested in decomposing the original signal in two terms, and amplitude term (that will determine the local energy) and a phase term. The analytic signal exactly performs this decomposition.

Given a unidimensional signal s(t) its analytic signal is defined by the complex function $s_{a}(t)=s(t)+{\mathrm{is}}_{H}(t)$, being $s_{H}(t)$ the Hilbert transform of s(t) an defined as $s_{H}(t)=1/\pi \int_{-\infty }^{\infty }\frac{s(t)}{t-\tau }d\tau$. Note that the Hilbert transform shifts the phase of the original signal in π/2. Thus, it is possible to define an amplitude terms as

(1) $$A(t)=\sqrt{s^{2}(t)+s_{H}^{2}(t)},$$

and a phase term

(2) $$\phi =\tan^{-1}\frac{s_{H}(t)}{s(t)},$$

which cast the signal decomposition $s_{a}(t)=A(t)e^{i\phi }$ as product of amplitude and phase. The extrapolation of the analytic signal to several dimension is named monogenic signal, and the amplitude of the analytic signal has its corresponding monogenic amplitude, that is the used in the *MonoRes/MonoTomo* algorithms, see (Vilas et al., 2018).

To illustrate the *MonoTomo* algorithm, a signal was constructed by concatenating signals of different frequencies (1/20,1/10 and 1/5rad/px), see left column red signal in Fig. 7. To be more realistic, Gaussian noise was added with zero mean and standard deviation of 0.08, blue curve. In the right column, the amplitude of the analytic signal is represented in blue, with a smoothed version in red, finally in green the amplitude of the analytic signal of noise is represented. Note how the monogenic amplitude is a step function showing that the energy of the wave is constant.

The *MonoRes* algorithm is divided in four steps: 1) high pass filtering of the signal; 2) determination of the monogenic amplitude (amplitude of the analytic signal in 1D); 3) smoothing of the monogenic amplitude; and 4) estimating the local resolution. The algorithm starts by high pass filtering the original signal from low to high frequencies, the resolution range is set by the user. In Fig. 7 second row, a high pass filter at 1/(20-∊) rad/px was performed, with ∊>0 a small number. The effect of this filter is that frequencies smaller than 1/(20-∊) rad/px are removed, and, indeed, it can be very clearly appreciated how the monogenic amplitude of the original synthetic signal in the part corresponding to a pure signal of 1/20 rad/px is almost absent. This can be better appreciated when a smoothing of the filtered signal is performed (in this case a low-pass filter of 1/(20-∊) rad/px has been used). Finally it remains to know which points of the filtered signal presents a local energy higher than the noise energy. To do so, the probability distribution of the monogenic amplitude of noise is then determined, followed by an hypothesis test to elucidate if the monogenic amplitude of the signal can be statistically distinguished of the noise. In the affirmative case, we will consider that point of the signal presents a resolution equal or higher than the filtering frequency. This process is repeated until a frequency is reached for which the amplitude of the monogenic signal cannot be distinguished from noise; this process can be followed in Fig. 7. However, a false positive can always happen, and in order to greatly reduce its occurrence we only consider that we reach the noise level when the statistical tests fails at that frequency and at the next one as well.
